# Coinfección por *Leishmania* spp. y *Trypanosoma cruzi* en *Didelphis marsupialis* (Mammalia: Didelphimorphia) del área urbana de Ovejas, Colombia

**DOI:** 10.7705/biomedica.7827

**Published:** 2026-06-01

**Authors:** Jorge Luis Rodríguez, Alveiro Pérez, Matilde Rivero, Carolina Ayala, Luis Gregorio Estrada, Samanta Cristina das Chagas, Eduar Elías Bejarano

**Affiliations:** 1 Grupo Investigaciones Biomédicas, Universidad de Sucre, Sincelejo, Colombia Universidad de Sucre Grupo Investigaciones Biomédicas Universidad de Sucre Sincelejo Colombia; 2 División de Investigación, Innovación y Desarrollo, Pyrogen S.A.S., Sincelejo, Colombia Pontificia Universidad Javeriana Pyrogen S.A.S Sincelejo Colombia; 3 Instituto Nacional de Medicina Tropical, ANLIS "Dr. Carlos G. Malbrán", Ministerio de Salud de la Nación, Puerto Iguazú, Argentina Instituto Nacional de Medicina Tropical, ANLIS "Dr. Carlos G. Malbrán" Ministerio de Salud de la Nación Puerto Iguazú Argentina; 4 Consejo Nacional de Investigaciones Científicas y Técnicas, Buenos Aires, Argentina Consejo Nacional de Investigaciones Científicas y Técnicas Consejo Nacional de Investigaciones Científicas y Técnicas Buenos Aires Argentina; 5 Laboratorio de Biología de Tripanosomátidos, Instituto Oswaldo Cruz, Río de Janeiro, Brasil Instituto Oswaldo Cruz Río de Janeiro Brasil

**Keywords:** leishmaniasis, enfermedad de Chagas, seroprevalencia, urbanización, zarigüeyas, Colombia, Leishmaniasis, Chagas disease, seroprevalence, urbanization, opossums, Colombia

## Abstract

**Introducción.:**

El municipio de Ovejas en Los Montes de María es un foco urbano autóctono de leishmaniasis y registra casos esporádicos de la enfermedad de Chagas. Aunque *Didelphis marsupialis* (zarigüeya) es reconocido como huésped, hospedador u hospedero, rural de tripanosomatídeos en Colombia, se desconoce su papel en esta zona del país.

**Objetivo.:**

Evaluar la frecuencia de infección natural por *Leishmania* spp. y *Trypanosoma cruzi* en *D. marsupialis* de ambientes peridomiciliarios del municipio de Ovejas.

**Materiales y métodos.:**

Entre agosto y octubre del 2015 se capturaron zarigüeyas en las áreas urbanas con trampas de vida Tomahawk. Tras evaluación morfométrica, identificación del sexo y conteo de piezas dentales, se obtuvieron muestras sanguíneas por punción cardiaca, usadas para detectar anticuerpos IgG contra *Leishmania* spp. y *T. cruzi,* amplificar blancos genéticos específicos de estos parásitos y para aislamiento *in vitro.* Los parásitos se tipificaron mediante análisis filogenético de los genes *citocromo b* y hsp70.

**Resultados.:**

Se capturaron 26 individuos (12 machos y 14 hembras; 16 adultos, 7 subadultos y 3 juveniles). Se obtuvo una prevalencia de anticuerpos del 23,08 y 53,85 %, contra *Leishmania* spp. y *T. cruzi,* respectivamente, con 11,54 % de coinfección. La frecuencia de infección molecular por *T. cruzi* fue del 61,54 y 23,08 % para *Leishmania* spp. Se logró aislar y genotipificar una cepa de *T. cruzi.*

**Conclusión.:**

Las altas frecuencias de infección simple y coinfección indican que *D. marsupialis* podría actuar como huésped sinantrópico de *Leishmania* spp. y *T. cruzi* en Ovejas, sugiriendo su participación en el mantenimiento de los ciclos domésticos de transmisión urbana.

## Abstract (en)

**Introduction.:**

The municipality of Ovejas, located in the Los Montes de María region, is an endemic focus of leishmaniasis and reports sporadic cases of Chagas disease. Although *Didelphis marsupialis* (the common opossum) is recognized as a rural reservoir of trypanosomatids in Colombia, its role in this area remains unknown.

**Objective.:**

To evaluate the natural infection frequency of *Leishmania* spp. and *T. cruzi* in *D. marsupialis* with *Leishmania* spp. and *T. cruzi* from peridomestic environments in Ovejas.

**Materials and methods.:**

Between August and October 2015, opossums were captured in urban areas using Tomahawk live traps. After morphometric evaluation, sex determination, and dental count, blood samples were obtained via cardiac puncture. These samples were used to detect IgG antibodies against *Leishmania* spp. and *T. cruzi,* amplify parasite-specific genetic targets, and perform *in vitro* isolation.

**Results.:**

A total of 26 individuals were captured (12 males and 14 females; 16 adults, 7 subadults, and 3 juveniles). Antibody prevalence was 23,08% for *Leishmania* and 53,85% for *T. cruzi,* with 11.54% co-infection. Molecular infection frequency was 61.54% for *T. cruzi* and 23,08% for *Leishmania* spp. One *T. cruzi* strain was successfully isolated and genotyped.

**Conclusion.:**

The high frequencies of single infection and co-infection suggest that *D. marsupialis* may act as a synanthropic host for *Leishmania* spp. and *T. cruzi* in Ovejas, indicating its potential role in maintaining domestic cycles of urban transmission.

La urbanización de las enfermedades parasitarias desatendidas es uno de los desafíos en salud pública más urgentes de los países en desarrollo ^1^^,^^2^. Este fenómeno está influenciado por diversas actividades humanas como la migración desde las áreas rurales a las urbanas, la deforestación y los cambios ambientales que modifican la ecología de los vectores y mamíferos huéspedes, hospedadores u hospederos, de algunos parásitos ^1^^,^^3^. Estos cambios han permitido que protozoos de la familia Trypanosomatidae (Euglenozoa: Kinetoplastea), incluyendo *Leishmania* spp., *Trypanosoma cruzi* y otros tripanosomas, extiendan su transmisión a los entornos urbanos, lo cual resulta en la aparición de casos en ciudades donde previamente no existían registros endémicos ^4^^-^^6^.

Tanto las leishmaniasis, causadas por protozoos del género *Leishmania,* como la enfermedad de Chagas, provocada por *T. cruzi,* afectan desproporcionadamente a las poblaciones vulnerables que residen cerca de las áreas forestales en zonas rurales o en asentamientos urbanos informales, en zonas de transición entre lo urbano y lo rural, que modifican la epidemiología de estas parasitosis y generan nuevas interacciones entre humanos, vectores, mamíferos huésped y ambiente ^1^^,^^7^.

En el ciclo zoonótico de estos parásitos, el humano es un huésped accidental, mientras que en la naturaleza circulan entre los mamíferos silvestres e insectos vectores específicos: dípteros de la familia Psychodidae para *Leishmania* spp. y hemípteros de la familia Reduviidae para *T. cruzi.* La distribución de estos parásitos está, por tanto, estrechamente ligada a la distribución geográfica de sus vectores y huéspedes dentro de ecosistemas específicos, los cuales están influenciados por variables ecológicas que varían en espacio y tiempo ^8^^,^^9^.

Algunos aspectos críticos en la epidemiología de ambas enfermedades son la sobreposición y los cambios en la distribución de los mamíferos hospederos de *Leishmania* spp. y *T. cruzi*^10^. Esta coincidencia geográfica no es infrecuente y puede llevar a fenómenos de coinfección en diferentes grupos de animales ^11^^-^^14^, lo que complica aún más su control. En este contexto, la detección de tripanosomatídeos en mamíferos es crucial para comprender los ciclos de transmisión y contribuir al desarrollo de estrategias eficaces de vigilancia epidemiológica y control ^12^, especialmente en áreas donde los límites entre ambientes domésticos y selváticos son difusos ^11^.

La detección de infección por tripanosomatídeos requiere de la integración de metodologías complementarias ^15^. Los métodos parasitológicos directos, como microscopía de tejidos y hemocultivo, permiten demostrar la presencia de formas viables del parásito y su potencial infectivo para los vectores ^16^^,^^17^. Por otro lado, los ensayos serológicos son valiosos para los estudios epidemiológicos, aun cuando presentan limitaciones de especificidad si no se emplean antígenos específicos estandarizados y dependen de la disponibilidad de controles de reacción y conjugados específicos para cada especie de interés ^18^^,^^19^. Finalmente, las técnicas moleculares ofrecen un método alternativo que brinda la posibilidad de determinar la identidad taxonómica de los parásitos infectantes ^18^^,^^20^^-^^22^.

La zarigüeya común, *Didelphis marsupialis* (Mammalia: Didelphimorphia), emerge como un actor clave en la epidemiología de estas enfermedades. En países como Brasil, Colombia, Venezuela y Panamá, se ha documentado ampliamente su importancia como huésped y amplificador de la transmisión de *Leishmania* spp. y *T. cruzi* en diversos escenarios ^10^^,^^23^^-^^27^. Este mamífero no solo es un reservorio natural de estos parásitos, sino que su comportamiento oportunista le permite colonizar áreas urbanas, facilitando la interacción entre los ciclos selváticos y domésticos ^27^^-^^29^.

Según los datos consolidados del Instituto Nacional de Salud, entre los años 2007 y 2023, el municipio de Ovejas, ubicado en la subregión de Los Montes de María, al norte de Colombia, registró la mayor incidencia de leishmaniasis en el departamento de Sucre, con 729 casos, de los cuales 688 (94,4 %) correspondieron a leishmaniasis cutánea, 5 casos (0,7 %) a leishmaniasis mucocutánea y 36 casos (4,9 %) a leishmaniasis visceral, constituyendo el segundo foco de leishmaniasis visceral más importante del país. Aunque la mayoría de los casos se presentaron en zonas rurales, también se registraron casos procedentes de áreas urbanas ^30^, donde fue posible demostrar la transmisión doméstica gracias a la identificación de infección natural en vectores y mamíferos domésticos ^31^^,^^32^.

Si bien Ovejas está categorizado como un municipio de riesgo bajo para la transmisión de la enfermedad de Chagas ^33^, en el 2024 se registró el primer caso autóctono en la cabecera municipal ^34^ y en un estudio previo se detectó ADN de *T. cruzi* en población humana ^35^. Además, en otras zonas del departamento de Sucre, muy similares en cuanto a zona de vida, proximidad geográfica y condiciones socioeconómicas, se han documentado brotes agudos de enfermedad de Chagas, como los ocurridos en el 2019 en el municipio El Roble (16 casos, de los cuales 2 fueron fatales) ^33^ y en el 2023 en San Marcos (15 casos) ^36^, lo que evidencia la necesidad de caracterizar los insectos vectores y los mamíferos huésped que mantienen los ciclos enzoóticos de estos parásitos en el departamento.

En la subregión de Montes de María, *D. marsupialis,* comúnmente conocida como «zorra», es una especie predominante ^37^, con una alta capacidad para adaptarse a los entornos urbanos. En las áreas peridomiciliarias de Ovejas, estos animales encuentran fuentes de alimento como desechos orgánicos, frutas y aves de corral, por lo cual son considerados como plaga por los habitantes.

Al considerar la presencia de zarigüeyas en la cabecera municipal de Ovejas y los antecedentes epidemiológicos de la transmisión de *Leishmania* spp. y *T. cruzi* en América, el objetivo de este estudio fue evaluar la frecuencia de infección natural por *Leishmania* spp. y *T. cruzi* en *D. marsupialis* de ambientes peridomiciliarios del municipio de Ovejas.

## Materiales y métodos

### Área de estudio

**Figura 1:**
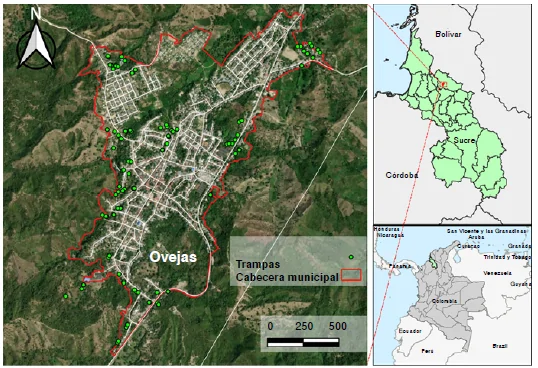
Distribución espacial del muestreo en la cabecera municipal de Ovejas (Sucre), al norte de Colombia

El estudio se llevó a cabo en la cabecera municipal de Ovejas en el departamento de Sucre, Colombia (figura 1). Este municipio pertenece al foco mixto de leishmaniasis de Montes de María, el principal foco de leishmaniasis visceral del Caribe colombiano. En términos ecológicos, la zona de vida corresponde a bosque seco tropical (Bs-T) ^38^, con temperatura media de 27,5 °C, humedad relativa de 77-80 % y precipitaciones de 1000-1300 mm por año.

### Tipo de estudio y captura de zarigüeyas

El estudio fue de tipo descriptivo y transversal. Se realizó un muestreo no probabilístico entre los meses de agosto y octubre del 2015. Los criterios de selección de las viviendas incluyeron: registros epidemiológicos de leishmaniasis y enfermedad de Chagas, con transmisión doméstica, presencia documentada de vectores, zonas verdes cercanas a las viviendas y avistamientos recurrentes de zarigüeyas. Se excluyeron viviendas que, aunque cumplían con estos criterios, no contaban con el consentimiento informado para la instalación de las trampas.

En las doce expediciones a campo, se visitaron 71 viviendas, distribuidas en 17/45 barrios de la cabecera municipal (37,78 %). En cada vivienda se instalaron de una a cuatro trampas de vida Tomahawk (66 x 23 x 23 cm; doce trampas noche). Las trampas con captura exitosa se retiraban y no se reemplazaban, mientras que las negativas eran reinstaladas por otra noche consecutiva.

Las trampas se instalaban en el peridomicilio (≤ 50 m de la vivienda), priorizando los sitios de avistamiento de zarigüeyas (por ejemplo, árboles frutales, posibles refugios y techos), con una separación mínima de 10 m lineales, cebadas con una banana madura *(Musa* sp.) e instaladas entre las 18:00 y las 06:00 horas. Los animales capturados fueron trasladados a un área de procesamiento en campo para su evaluación física y la toma de muestras.

### *Examen f**í**sico y toma de muestras biológicas*

Bajo anestesia inducida por la inyección intramuscular de una mezcla de ketamina y xilacina (30 mg/kg y 1 mg/kg, equivalentes a 0,3 ml y 0,05 ml por cada kilogramo de peso, respectivamente), las zarigüeyas fueron examinadas en búsqueda de signos clínicos sugestivos de infección por tripanosomatídeos (lesiones cutáneas, alopecia, conjuntivitis u otras alteraciones evidentes). Se registró el sexo, se verificó la presencia de crías en el marsupio de las hembras y se estimó la edad con base en el número de piezas dentales emergidas en la hilera maxilar izquierda ^39^^,^^40^.

Mediante punción cardíaca se obtuvieron entre 3 y 5 ml de sangre (se usaron jeringas de 5 ml y agujas de 11/ pulgada calibre 22 en adultos y subadultos, y jeringas de 3 ml con agujas de 1 pulgada, calibre 23 para juveniles). Las muestras de sangre se distribuyeron en tubos sin aditivo para la obtención del suero mediante centrifugación a 3000 revoluciones por minuto (r.p.m.) y tubos heparinizados para la extracción de ADN. Tres muestras de sangre de las últimas dos expediciones se destinaron para el aislamiento parasitológico. Posteriormente, se implantó un microchip de radio frecuencia subcutáneo en la región cervical.

Después de finalizar los procedimientos de la toma de muestras, cada zarigüeya fue colocada en su respectiva jaula, en un espacio ventilado, protegidas contra la insolación (mediante cobertura con hojas frescas de árboles cercanos) y provistas de agua y alimento balanceado para conejos *ad libitum.* Todos los individuos fueron liberados al anochecer en áreas alejadas de su sitio de captura, únicamente después de verificar su completo estado de recuperación fisiológica y conductual, evidenciados por su activa movilidad y por la salida voluntaria de la jaula.

### Cultivo in vitro de tripanosomatídeos

Para el aislamiento en cultivo de los parásitos, se inocularon ~200 μl de sangre en un medio de cultivo bifásico compuesto por una fase sólida constituida por el medio Novy, McNeal y Nicolle (NNN) y una fase líquida constituida por el medio Roswell Park Memorial Institute 1640 (RPMI 1640), suplementado con suero fetal bovino (10 %), penicilina (10 000 U/ml), estreptomicina (100 000 mg/ml) y 5-fluorocitosina (50 μg/ml).

Los cultivos se incubaron a 26 °C y se examinaron semanalmente durante 90 días bajo un microscopio de luz invertido a 40X, para detectar crecimiento de tripanosomatídeos.

### Diagnóstico serológico de Leishmania spp. y Trypanosoma cruzi por inmunofluorescencia indirecta

Los sueros obtenidos de las muestras de sangre se almacenaron a -20 °C hasta su análisis. La detección de anticuerpos IgG contra *Leishmania* spp. y *T. cruzi* se realizó mediante inmunofluorescencia indirecta (IFI), utilizando como antígenos a los promastigotes de *Leishmania braziliensis* y *Leishmania infantum,* propagados en medio Schneider, y epimastigotes de *T. cruzi* en fase exponencial de crecimiento, cultivados en el medio *Liver Infusion Tryptose* (LIT), todos fijados en placas. Para garantizar la especificidad de las reacciones, se incluyeron como controles positivos sueros de *D. marsupialis* infectados experimentalmente con *Leishmania* spp. y *T. cruzi,* y como controles negativos, sueros de zarigüeyas libres de agentes patógenos confirmadas por IFI y por reacción en cadena de la polimerasa (PCR).

En las reacciones se incluyó un anticuerpo intermedio anti-IgG de zarigüeyas producido en conejos y un conjugado de isotiocianato de fluoresceína anti-IgG de conejo, diluido en azul de Evans 1X. Como punto de corte se estableció la dilución 1:40 ^41^^,^^42^.

Se consideró que una muestra era positiva para *Leishmania* spp. a partir del punto de corte, solo cuando el título para *T. cruzi* estaba por debajo de 1:40 y viceversa. Se consideró coinfección cuando los títulos para ambos parásitos fueron iguales a 1:80 o superiores. Se consideraron reacciones cruzadas cuando ambos títulos fueron iguales al punto de corte.

### Extracción de ADN genómico de los parásitos

La extracción del ADN genómico se realizó a partir de 200 μl de sangre heparinizada y de los parásitos aislados en cultivo, usando el kit comercial QIAamp™ DNA Mini QIAcube, en un equipo automatizado de extracción de ácidos nucleicos (QIAcube™ Robotic Workstation), siguiendo los protocolos recomendados por el fabricante. La concentración final y la pureza de los extractos de ADN de cada muestra se determinaron en un espectrofotómetro (Nanodrop 2000™ Thermo Scientific). Los extractos de ADN se almacenaron a -20 °C hasta su análisis.

### Detección de Leishmania spp. y Trypanosoma cruzi mediante PCR

La detección inicial de los parásitos se realizó mediante dos enfoques de PCR. Para *Leishmania* spp., se amplificó por PCR convencional la región conservada del minicírculo del kinetoplasto (ADNk) usando los cebadores 13A/13B que delimitan un fragmento de 120 pares de base ^43^. Por otro lado, para la detección de *T. cruzi,* se amplificó una secuencia repetitiva de microsatélite nuclear de 188 pb con los cebadores específicos TCZ1/TCZ2 ^44^.

Todas las reacciones de amplificación por PCR se realizaron en un volumen final de 25 μl que contenía: 1 U de ExAct^TM^ Plus Taq DNA Polymerase (Mbiotech, Inc.), mezcla de reacción 1X con dNTP a 0,2 mM, 0,24 pmol de cada cebador y entre 10-100 ng de ADN molde. El perfil térmico para cada reacción se ajustó a las condiciones recomendadas por el fabricante de la polimerasa y a la temperatura de alineamiento de cada pareja de cebadores (cuadro 1). Como controles positivos se usaron ADN de una cepa local de *L. infantum* (MHOM/CO/2013/133), aislada de un paciente con leishmaniasis visceral, y ADN de *T. cruzi;* se usó agua ultrapura como control de reacción [control sin planilla (no *template control,* NTC)].

**Cuadro 1:** Condiciones de PCR para detección y tipificación de tripanosomatídeos

Blanco genético	Cebador	Secuencia 5' - 3'	Perfil térmico				Tamaño (pb)	Ref.
			Paso de la reacción		Temperatura	Tiempo		
					(°C)	(s)		
Región conservada del minicírculo	13A	GTG GGG GAG GGG CGT TCT	Desnaturalización inicial		95	120	120	(31)
			x 35 ciclos	Desnaturalización	95	20		
				Alineamiento	53,8	40		
	13B	ATT TTA CAC CAA CCC CCA GTT		Extensión	72	12		
			Extensión final		72	300		
Secuencias repetitivas de microsatélites (ADNn)	TCZ1	CGA GCT CTC TTG CCC ACA CAC GGG TGC T	Desnaturalización inicial		95	120	188	(32)
			x 35 ciclos	Desnaturalización	95	20		
				Alineamiento	57	40		
	TCZ2	CCT CCA AGC AAG CAG CGG ATA GTT CAG G		Extensión	72	20		
			Extensión final		72	300		
Gen citocromo b	LCBF1	GGT GTA GGT TTT AGT YTA GG	Desnaturalización inicial		95	120	860	(33,34)
			x 35 ciclos	Desnaturalización	95	20		
				Alineamiento	54	40		
	LCBR2M	ACA ATA AAC AAA TCA TAA TAT RCA ATT		Extensión	72	60		
			Extensión final		75	300		
	CB Cytb F	AAG TGG TGT YTG YTT AGC RTG	Desnaturalización inicial		95	120	728	(35)
			x 35 ciclos	Desnaturalización	95	20		
				Alineamiento	51	40		
	CB Cytb B	GGSACTGCTTTW ARR AAG CC		Extensión	72	60		
			Extensión final		72	300		
Gen hsp70	hsp70sen	GAC GGT GCC TGC CTA CTT CAA	Desnaturalización inicial		95	120	1422	(36,37)
			x 35 ciclos	Desnaturalización	95	20		
				Alineamiento	59	60		
	hsp70ant	CCG CCC ATG CTC TGG TAC ATC		Extensión	72	120		
			Extensión final		72	480		

5'-3': sentido de las secuencias de nucleótidos; ADNn: ADN nuclear; Ref.: referencia bibliográfica

Los productos amplificados fueron separados por electroforesis en geles de agarosa al 1,5 %, teñidos con GelStar™, visualizados y documentados en un equipo Quantum-ST4™ (Vilber Lourmat).

### Tipificación de los parásitos mediante PCR, secuenciación y análisis filogenético

Para la identificación específica se realizó una PCR anidada del gen *citocromo b (Cyt-b).* En la primera ronda de reacción se usaron los cebadores LCBF1/LCBR2M ^45^ modificados a partir de los propuestos por Luyo-Acero y colaboradores ^46^, los cuales delimitan un fragmento de 860 pb.

En la segunda ronda, se usaron los cebadores CB Cytb F/CB Cytb B que delimitan un fragmento intermedio de 728 pb ^47^. Además, se amplificó un fragmento del gen nuclear de la proteína de choque térmico de 70 kD (hsp70, *Heat Shock Protein* 70), usando los cebadores hsp70sen/hsp70ant ^48^^,^^49^, que delimitan un fragmento de ~1422 pb de la región codificante de este gen. En el cuadro 1 se muestra el listado de los cebadores usados, con sus correspondientes secuencias nucleotídicas.

Los productos positivos fueron secuenciados por el método de Sanger ^50^^).^ Los electroferogramas obtenidos con cada cebador fueron editados en el programa MEGA 5.0 ^51^ para descartar ambigüedades en cada posición y construir secuencias en consenso que luego fueron comparadas en el GenBank del *National Center for Biotechnology Information* (NCBI) mediante el uso de la herramienta de búsqueda de alineamiento local BLASTn *(Basic Local Alignment Search Tool for nucleotides)*^52^, con el fin de hacer un acercamiento inicial de la especie detectada. La identidad específica se confirmó mediante la construcción de dendrogramas a partir de secuencias homólogas de cada gen, con el programa MEGA 5.0 ^51^.

### Análisis de datos

La seroprevalencia de anticuerpos IgG contra *Leishmania* spp. y *T. cruzi* se calculó como el porcentaje de zarigüeyas positivas por IFI frente al total muestreado.

Para la frecuencia de infección molecular, se consideraron las muestras positivas por PCR (PCR-ADNk para *Leishmania* spp. y PCR-TCZ para *T. cruzi)* divididas entre el total de individuos analizados.

La coinfección se determinó como la proporción de individuos simultáneamente positivos para ambos agentes patógenos tanto por IFI como en ambas PCR específicas.

Las asociaciones entre variables demográficas (edad y sexo) y el estado de infección (por IFI y PCR) se analizaron mediante pruebas de X^2^ y pruebas exactas de Fisher para proporciones múltiples (α = 0,05) utilizando el *software* R, versión 4.4.1. ^53^.

### Consideraciones éticas

El estudio contó con la aprobación del Comité Institucional de Bioética de la Universidad de Sucre (Acta N° 02/2012) para el proyecto «Incriminación de reservorios de *Leishmania* spp. en un foco urbano de leishmaniasis cutánea de los Montes de María, Costa Caribe colombiana». Para el acceso a las viviendas participantes se contó con el consentimiento informado por escrito de los jefes de hogar. Todos los procedimientos cumplieron la normativa colombiana, incluyendo el Estatuto Nacional de Protección de los Animales (Ley 84 de 1989) ^54^, actualizado por la Ley 2455 de 2025 (Ley Ángel), así como la Resolución 8430 de 1993 (Artículo 87) ^55^ para investigación en salud. Todos los ejemplares fueron liberados en su hábitat tras confirmar su recuperación posanestésica (movilidad normal) y demás procedimientos. Ningún espécimen liberado mostró signos de deterioro atribuible a los procedimientos.

## Resultados

### Esfuerzo de captura y éxito de muestreo

**Figura 2:**
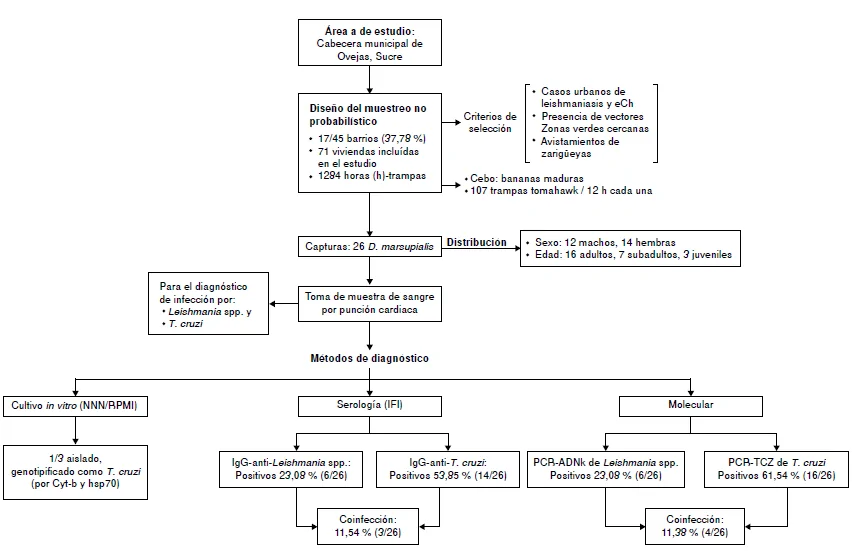
Diagrama de flujo integral del estudio en el que se resume la secuencia metodológica: incluye el diseño del muestreo, los criterios de selección de las viviendas y los métodos de diagnóstico; asimismo se detalla el número de zarigüeyas capturadas, su distribución por sexo y edad, y los resultados de frecuencias de infección y coinfección por las técnicas de diagnóstico aplicadas (cultivo, IFI y PCR).

**Figura 3:**
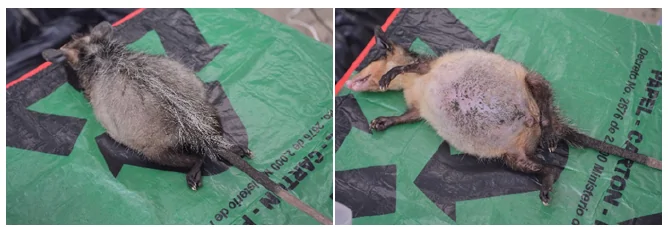
Signos clínicos en espécimen hembra de *D. marsupialis* con dilatación abdominal y pérdida de peso corporal (ID: 18 en el cuadro 2).

El esfuerzo de muestreo totalizó 1284 horas (107 trampas instaladas durante 12 horas cada una), resultando en una tasa de captura del 24,30 % y una infestación domiciliaria del 28,17 % (20/71 viviendas). Se capturaron 26 zarigüeyas con la siguiente distribución demográfica: adultos, 61,5 % (n = 16); subadultos, 27,0 % (n = 7), y juveniles, 11,5 % (n = 3), mientras que la proporción sexual mostró un ligero predominio de hembras (54 %, 14/26) sobre machos (46 %, 12/26). La figura 2 integra de manera esquemática la metodología global y los hallazgos derivados del muestreo, observándose dos hembras con crías en el marsupio (cuadro 2). Solo un individuo presentó alteraciones físicas notables (dilatación abdominal y pérdida de peso corporal; figura 3), sin otros signos clínicos asociados a infección por tripanosomatídeos.

Nota: en rojo se resaltan los datos obtenidos del individuo con dilatación abdominal; en negrita se resalta el individuo a partir del cual se aislaron los parásitos en cultivo

**Cuadro 2:** Caracterización de infecciones por *Leishmania* spp. y *Trypanosoma cruzi* en los especímenes de *Didelphis marsupialis* del municipio de Ovejas (Sucre), norte de Colombia

ID	Sexo	Crías	Grupo etario	Serología			PCR				Cultivo
				Título *Leishmania*	Título *T. cruzi*	Diagnóstico	*Leishmania* (ADNk)	*T. cruzi* (TCZ)	Cit b	hsp70	Medio NNN/RPMI
		
1	H	0	Adulto	1:80	1:20	*Leishmania_+*	-	+	-	+	
2	M		Subadulto	1:40	1:80	*T. cruzi_+*	-	+	-	+	
3	M		Subadulto	1:10	1:40	*T. cruzi_+*	-	+	-	+	
4	H	0	Adulto	1:20	1:40	*T. cruzi_+*	-	+	-	-	
5	H	0	Subadulto	1:20	1:40	*T. cruzi_+*	-	+	-	+	
6	M		Juvenil	-	-	negativo	-	-	-	-	
7	M		Adulto	-	-	negativo	+	+	-	-	
8	H	7	Adulto	1:40	1:10	*Leishmania_+*	-	+	-	-	
9	M		Adulto	1:10	1:40	*T. cruzi_+*	-	-	-	+	
10	H	4	Adulto	-	-	negativo	+	-	-	+	
11	H	0	Adulto	1:10	1:40	*T. cruzi_+*	+	+	-	+	
12	H	0	Subadulto	1:20	1:20	negativo	-	-	-	-	
13	H	0	Subadulto	1:20	1:40	*T. cruzi_+*	-	-	-	-	
14	M		Adulto	1:40	1:40	Rx_cruzada	+	-	-	-	
15	H	0	Adulto	1:10	1:40	*T. cruzi_+*	-	-	-	+	
16	H	0	Juvenil	1:40	1:80	*T. cruzi_+*	-	+	-	-	
17	M		Juvenil	1:10	-	negativo	-	-	-	-	
18	H	0	Subadulto	1:40	1:40	Reacción_cruzada	-	-	-	-	
19	M		Subadulto	1:20	1:40	*T. cruzi_+*	-	+	-	-	
20	H	0	Adulto	1:160	1:320	Coinfección	+	+	-	+	
21	M		Adulto	1:40	1:40	Rx_cruzada	-	-	-	+	
22	M		Adulto	1:80	1:160	Coinfección	-	+	-	+	-
23	M		Adulto	1:20	1:40	*T. cruzi_+*	+	+	-	-	-
24	H	0	Adulto	1:80	1:160	Coinfección	-	+	+	+	+
25	M		Adulto	1:40	1:40	Rx_cruzada	-	+	-	-	
26	H	0	Adulto	1:80	1:10	*Leishmania_+*	-	+	-	-	

Resultados individuales de serología (IFI), PCR y cultivo para *Leishmania* spp. y *Trypanosoma cruzi* en *Didelphis marsupialis* de Ovejas (Sucre).

ID: código de ingreso en la base de datos; H: hembra; NNN: Novy-MacNeal-Nicolle; M: macho; (+): resultado positivo; (-): resultado negativo; RPMI: *Roswell Park Memorial Institute*

### Aislamiento parasitológico en cultivo

De las tres muestras sanguíneas inoculadas en medio de cultivo, una mostró crecimiento de parásitos uniflagelados, que posteriormente fueron caracterizados como *T. cruzi.*

### *Seroprevalencia de Leishmania* spp. *y Trypanosoma cruzi por IFI*

El análisis por IFI reveló que el 80,77 % (21/26) de los sueros evaluados presentaron reactividad, al menos, para uno de los dos parásitos evaluados. La distribución de los patrones de reactividad mostró que 11,54 % (3/26) fueron exclusivamente positivos para *Leishmania* spp., 42,31 % (11/26) positivos para *T. cruzi* solamente, 11,54 % (3/26) con coinfección y 15,38 % (4/26) fueron reacciones cruzadas. Las seroprevalencias globales fueron significativamente mayores para *T. cruzi* (53,85 %) que para *Leishmania* spp. (23,08 %) (cuadro 2).

### Detección molecular de Leishmania spp. y Trypanosoma cruzi por PCR

El análisis molecular mediante PCR específica reveló una frecuencia de positividad del 23,08 % (6/26) para *Leishmania* spp. (usando el marcador ADNk) y del 61,54 % (16/26) para *T. cruzi* (con el marcador TCZ). Se detectó infección con ambos parásitos en el 15,38 % (4/26) de los individuos, es decir, resultaron positivos por ambos marcadores genéticos (cuadro 2).

### Tipificación molecular y análisis filogenético

La cepa de parásitos tripanosomatídeos aislada en cultivo mostró amplificación positiva mediante la PCR anidada del gen *Cyt-b,* aunque este marcador no amplificó en las muestras sanguíneas directas. En contraste, la PCR-hsp70 detectó infección en doce muestras sanguíneas (46,15 %) y generó un amplicón adicional correspondiente al aislado de cultivo (cuadro 2).

**Figura 4:**
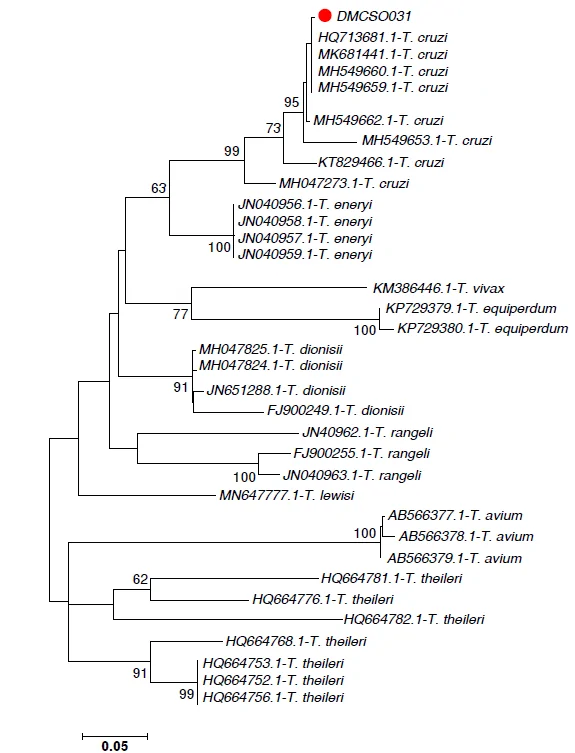
Filogenia de *Trypanosoma cruzi* basada en el gen *citocromo b.* Dendrograma construido empleando el método *neighbor-joining* (las distancias genéticas se calcularon mediante el método Tamura de 3 parámetros) de comparaciones pareadas del gen Citocromo b de una cepa de *Trypanosoma cruzi* aislada de una zarigüeya común en el área urbana del municipio de Ovejas (círculo rojo). En la base de cada grupo se muestran los soportes de ramas inferidos a partir de 10 000 réplicas. Se incluyeron en el análisis 33 secuencias nucleotídicas homólogas, disponibles en la base de datos de nucleótidos de GeneBank, cuyos números de acceso aparecen codificados en las etiquetas de cada secuencia, y se compararon 387 posiciones.

La secuenciación de los amplicones derivados de la cepa aislada por cultivo generó secuencias consenso de *Cyt-b* (702 pb) y hsp70 (1313 pb). Los análisis de similitud mediante BLASTn revelaron una identidad del 99 % o más, cobertura del 99 % y valores E = 0,0 contra secuencias de referencia de *T. cruzi* en ambas regiones genómicas. Los análisis filogenéticos confirmaron la agrupación de estas secuencias dentro del clado de *T. cruzi,* con soportes de *boostrap* del 95 % *(Cyt-b)* y del 99 % (hsp70) (figuras 4 y 5, respectivamente).

**Figura 5:**
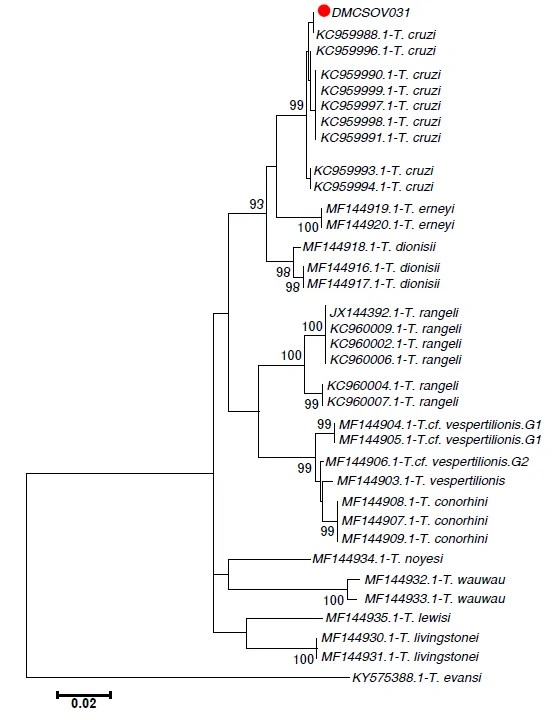
Filogenia de *Trypanosoma cruzi* basada en el gen de la hsp70. Dendrograma construido empleando el método *neighbor-joining* (las distancias se calcularon mediante el método Tamura-Nei) de comparaciones pareadas del gen de la hsp70 de una cepa de *Trypanosoma cruzi* aislada de una zarigüeya común en el área urbana del municipio de Ovejas (círculo rojo). En la base de cada grupo se muestran los soportes de ramas inferidos a partir de 10 000 réplicas. Se incluyeron en el análisis 34 secuencias nucleotídicas homólogas, disponibles en la base de datos de nucleótidos de GeneBank, cuyos números de acceso aparecen codificados en las etiquetas de cada secuencia, y se compararon 626 posiciones.

En contraste, los electroferogramas de los doce amplicones de hsp70, derivados de muestras sanguíneas, mostraron señales mezcladas, lo cual sugiere amplificación inespecífica, puesto que once secuencias tuvieron cierto grado de similitud con el genoma de *Monodelphis domestica,* mientras que una mostró semejanza con secuencias del género *Trypanosoma,* lo que impidió realizar análisis filogenéticos confiables con estos datos.

### Análisis estadístico

No se encontraron asociaciones estadísticas (p > 0,05) entre la infección por *Leishmania* spp. y *T. cruzi* (por IFI o PCR), con las variables sexo y edad (cuadro 3).

**Cuadro 3:** Pruebas de asociación estadística entre los resultados de detección de *Leishmania* spp. y *Trypanosoma cruzi* respecto al sexo y edad de zarigüeyas del área urbana del municipio de Ovejas

Variable	Categoría	n	Prueba estadística	IFI (+)				PCR (+)		
				*Leishmania*	p	*T. cruzi*	p	ADNk	p TCZ	p
Sexo	Hembra	14	X^2^	5	0,1696	8	0,7157	2	0,3652 9	1
	Macho	12		1		6		4	7	
Grupo etario	Adulto	16	Prueba exacta de Fisher	6	0,1341	8	0,5243	2	0,2399 11	0,611
	Subadulto	7		0		5		3	4	
	Juvenil	3		0		1		1	1	

## Discusión

En el contexto epidemiológico, las altas tasas de infección por *Leishmania* spp. (serología: 23,08 %; PCR: 23,08 %) y *T. cruzi*(serología: 53,85 %; PCR: 61,54 %), junto con una coinfección de 11,54 % y el aislamiento exitoso de *T. cruzi,* representan las primeras líneas de evidencia para demostrar que *D. marsupialis* del área urbana de Ovejas cumple con los criterios de incriminación descritos por la Organización Mundial de la Salud (OMS) ^56^. Además, la ausencia de signos clínicos y la distribución homogénea de infección entre sexos y grupos etarios sugieren un equilibrio dinámico entre *D. marsupialis* y estos parásitos, que es necesario para que una especie pueda asegurar su mantenimiento por largos periodos ^28^.

Este equilibrio dinámico, en conjunto con el comportamiento sinantrópico de *D. marsupialis,* puede ayudar a comprender el papel potencial de esta especie como puente epidemiológico entre los ciclos selváticos y domésticos en la urbanización de ambas enfermedades en Ovejas, como ha ocurrido en contextos epidemiológicos similares en Brasil ^57^, y explicaría en parte el hallazgo de *Leishmania* spp. en humanos, perros y vectores ^31^^,^^32^^,^^58^ residentes en la cabecera municipal de Ovejas. Asimismo, justifican el fracaso de las estrategias de control basadas exclusivamente en vectores y sacrificio canino, como lo evidencia el registro de casos nuevos de leishmaniasis cutánea en el 2024 ^59^.

El aislamiento de *T. cruzi* en cultivo indica una fase aguda de infección con parasitemia periférica, lo que implica la alta biodisponibilidad para vectores. Aunque no se evaluó la presencia de triatominos, la elevada frecuencia de infección podría estar asociada a los hábitos carnívoros de las zarigüeyas, la posible transmisión fecal-oral o la transmisión vertical ^41^^,^^60^^,^^62^, pero se necesitan estudios futuros para dilucidar los mecanismos de transmisión predominantes.

Las frecuencias de infección por *Leishmania* spp. y *T. cruzi* registradas en *D. marsupialis* en las Américas varían entre el 3 y el 80 % ^10^^,^^23^^,^^26^^,^^27^^,^^41^^,^^63^^-^^75^. En Colombia, se observaron resultados similares en la zona metropolitana de Bucaramanga ^76^, Montes de María ^69^ y Cartagena durante un brote autóctono urbano de leishmaniasis visceral ^6^. En este sentido, los antecedentes epidemiológicos de *D. marsupialis* demuestran consistentemente su participación como huésped en la transmisión urbana de la leishmaniasis y de la enfermedad de Chagas.

El resultado de seroprevalencia de infección por *T. cruzi* (53,85 %) refleja la ocurrencia de un ciclo enzoótico urbano, sin que esto implique necesariamente la ocurrencia de casos de enfermedad de Chagas en la población humana ^10^. Sin embargo, este resultado ayuda a comprender el hallazgo de ADN parasitario en pacientes que aparentemente habían sufrido síntomas consistentes con esta enfermedad después de haber sido picados por triatominos en las viviendas de áreas urbanas y rurales de este municipio ^35^. Esto sugiere un subdiagnóstico y un riesgo mayor al estimado oficialmente.

La coinfección de *Leishmania* spp. y *T. cruzi* (11,54 %) encontrada en este estudio supera la reportada para *T. cruzi* y *T. rangeli* en el área urbana de Bucaramanga (1,43 %) ^76^, pero es inferior a la coinfección de *Leishmania* spp. y *T. cruzi* registrada en áreas rurales y urbanas de Venezuela (14,3 %) ^24^ y a la coinfección de *T. cruzi* y *T. rangeli* registrada en los focos selváticos panameños (15,79 %). Estas diferencias podrían atribuirse a variables metodológicas, ecológicas y sociosanitarias.

La coexistencia de vectores en el peridomicilio podría ayudar a explicar el solapamiento de ciclos silvestres y domésticos. Así, para el caso de la enfermedad de leishmaniasis, se han documentado poblaciones de *Lutzomyia evansi*^77^.

Para reducir el riesgo epidemiológico que representa la presencia de zarigüeyas en la zona urbana de Ovejas, se recomienda un manejo con enfoque integral que tenga en cuenta su papel ecológico, los riesgos zoonóticos y el contexto local, considerando estrategias que incluyan la modificación del hábitat mediante la reducción de disponibilidad alimenticia (manejo estricto de residuos orgánicos) y la eliminación de refugios (sellado de estructuras), combinado con exclusión física con mallas metálicas. También se deben incluir medidas preventivas no letales como repelentes de olor ^78^, aspersores activados por movimiento y dispositivos ultrasónicos, control integrado de vectores con fumigación residual para triatominos y manejo ambiental para flebotomíneos, remoción ética mediante captura de especímenes vivos y reubicación a cargo de profesionales cuando sea necesario, dando prioridad a la vigilancia con diagnóstico molecular avanzado (PCR multiblanco y antígenos recombinantes) para detectar infecciones simples y coinfecciones. Esta estrategia articula sostenibilidad ecológica y seguridad sanitaria.

Por otra parte, la PCR-ADNk permitió la detección del material genético de parásitos en el 23,08 % (6/26) de las muestras analizadas; sin embargo, la tipificación mediante *Cyt-b* y hsp70 no fue posible, debido a la amplificación inespecífica del ADN del huésped. Estos resultados contrastan con el desempeño analítico de ADNk y hsp70 usados en conjunto en muestras biológicas de roedores en Brasil, en los que ambos marcadores permitieron la identificación de los subgéneros *Leishmania* y *Viannia,* incluso en muestras con baja carga parasitaria ^79^, lo cual resalta la necesidad de validar blancos genéticos moleculares específicos cuando se trabaja con marsupiales.

La ausencia de signos clínicos asociados a la infección con ambos grupos de parásitos tripanosomatídeos en *D. marsupialis* contrasta con el hallazgo registrado para el área metropolitana de Bucaramanga, donde se encontró que el 50 % de las zarigüeyas analizadas presentaban signos atribuibles a la infección por *T. cruzi*^76^. Este contraste se puede explicar por las diferencias en recursos alimenticios, virulencia de los parásitos o el estado de infección. Asimismo, el estado de salud de las zarigüeyas capturadas en Ovejas sugiere infecciones subclínicas, como se ha descrito en estudios de especímenes asintomáticos infectados con *L. infantum* y *T. cruzi,* cuyas necropsias han revelado megalias y alteraciones moderadas en hígado, nódulos linfáticos, bazo, corazón, aparato digestivo y cerebro ^80^^-^^82^.

En conclusión, este estudio evidencia que *D. marsupialis* estaría potencialmente actuando como huésped sinantrópico en el mantenimiento de las poblaciones urbanas de *Leishmania* spp. y *T. cruzi* en Ovejas, sustentado por las altas frecuencias de infección (23,08 y 61,54 %, respectivamente) y coinfección (11,54 %), junto con el aislamiento de *T. cruzi.*

No obstante, el estudio presentó limitaciones metodológicas que deben considerarse. En primer lugar, el tamaño de la muestra (n = 26) restringe la potencia estadística para hacer inferencias por sexo y edad a nivel poblacional. En segundo lugar, la estrategia de captura mediante cebo frutal (banana madura) podría introducir un sesgo de selección hacia individuos con preferencias tróficas frugívoras. No obstante, esta decisión metodológica se justificó tras un estudio piloto que demostró que el uso de cebos animales (vísceras de pollo) generaba capturas incidentales de gatos domésticos (datos no mostrados), lo que representaba un riesgo potencial para la integridad de las zarigüeyas y alteraba el objetivo de muestreo. En tercer lugar, los marcadores moleculares empleados *(Cyt-b* y hsp70) mostraron baja especificidad para tipificar *Leishmania* spp. debido a interferencia del ADN de este mamífero. Estas restricciones resaltan la necesidad de validar dianas genéticas específicas para marsupiales en estudios futuros.

Finalmente, se recomienda que, para mitigar el riesgo zoonótico, se deben reorientar las estrategias de control hacia: 1) la implementación de diagnóstico integrado con PCR multiblanco y antígenos recombinantes para discriminar coinfecciones, 2) intervenciones diferenciadas contra vectores (fumigación residual para triatominos y manejo ambiental y del domicilio para flebotomíneos) y 3) reducción de sinantropía mediante barreras físicas que impidan la entrada de estos animales a las viviendas y manejo de residuos orgánicos. Estas acciones podrían interrumpir los ciclos domésticos de transmisión identificados.
